# Ocular surface heat flux density as a biomarker related to diabetic retinopathy (pilot study)

**DOI:** 10.1016/j.aopr.2024.03.004

**Published:** 2024-03-26

**Authors:** Lukyan Anatychuk, Roman Kobylianskyi, Oleg Zadorozhnyy, Taras Kustryn, Illia Nasinnyk, Andrii Korol, Nataliya Pasyechnikova

**Affiliations:** Institute of Thermoelectricity of the National Academy of Sciences of Ukraine and the Ministry of Education and Science of Ukraine, Chernivtsi, Ukraine; Yuriy Fedkovych Chernivtsi National University, Chernivtsi, Ukraine; State Institution “The Filatov Institute of Eye Diseases and Tissue Therapy of the National Academy of Medical Sciences of Ukraine”, Odesa, Ukraine

## Dear editor:

Thermoregulation ensures a constant body temperature and is critical for maintaining homeostasis in the human body.^1^ In healthy individuals, body temperature is well-balanced and fluctuates within a narrow range.^2^ The normal continuous course of metabolic reactions in the human body and its vital activity in various conditions depends on the temperature equilibrium.^3^

Body temperature is one of the most important measurable criteria for assessing the functioning of the human body, along with heart rate, blood pressure and respiratory rate.^4^ Temperature as a physiologic biomarker is widely used in clinical practice to diagnose and monitor various diseases.^5^ However, for a complete understanding of the intensity of heat transfer, it is advisable to measure the temperature values along with a quantitative assessment of heat flux (HF). This helps to improve the understanding of the functioning of organs and tissues. It is known that the presence of a temperature gradient in the human eye (between the choroid, which is the main source of heat, and the cornea, which is in contact with the environment) generates HF, directed from a region of higher temperature to a region of lower temperature. This HF can be detected and measured on the outer ocular surface.^6^

The heat transfer features in the body of patients with diabetes mellitus, which includes the organ of vision, are not yet fully understood.^7^ Patients with diabetes are likely to have thermoregulatory dysfunction, which is manifested by impaired heat removal.^7^^,^^8^ It increases the risk of heat stress-related diseases.^9^ Other authors also report features of foot skin and ocular surface temperature (OST) in patients with diabetes that can be used for diagnosis and monitoring. According to Papanas et al., individuals with type 2 diabetes and peripheral neuropathy had notably higher foot temperatures than those without neuropathy,^10^ which is likely due to the phenomena of subclinical inflammation. Chandrasekar et al. recorded a lower OST in patients with diabetic retinopathy (DR) compared to healthy individuals,^11^ which, apparently, is due to circulatory disorders in the choroid. We hypothesize that HF detected and quantified on the ocular surface, along with temperature, can be successfully used as a physiological biomarker for diagnosing ocular heat transfer status and predicting ocular manifestations of diabetes.

In our previous studies, we presented the first results of measurements of the HF density of the eyes of experimental animals using an original thermoelectric device.^12^ When assessing heat transfer in healthy individuals, differences were revealed in the values of the ocular HF depending on the person's age.^13^ A correlation was found between the thickness of the choroid and the HF density of the eyes of healthy persons.^14^ Thus, the present study aimed to evaluate ocular surface heat flux density in patients with diabetic retinopathy.

## 1 Material and methods

### 1.1 Study design

This was a pilot, prospective, open-label and non-interventional study. Eighty-four patients (168 eyes; aged 18–88 years) with diabetes mellitus and DR were under our observation. The study included patients with the same stage of DR in both eyes. Exclusion criteria were: neovascular glaucoma; intraocular surgery (except uncomplicated cataract surgery carried out more than 3 months before the start of the study); the presence of acute intraocular or periocular inflammatory processes. Thirty healthy volunteers (60 eyes; aged 18–85 years) were also observed.

All study participants underwent bilateral ophthalmic examination including biomicroscopy, ophthalmoscopy, optical coherence tomography, and measurements of the ocular surface temperature and HF density. Optical coherence tomography was performed to determine the retinal thickness and the subfoveal thickness of the choroid (Spectralis, Heidelberg Engineering, Germany).

### 1.2 The device for measurement of the ocular surface HF density and the OST

A multichannel thermoelectric device, originally designed for ophthalmology research, was utilized to determine the density of HF and OST.^13^ This device consists of a thermoelectric HF sensor and an electronic recording unit.

A small-sized thermoelectric HF sensor for use in this device was developed and made according to the technology of the Institute of Thermoelectricity of the National Academy of Sciences of Ukraine. A thermoelectric miniature module (2 mm ​× ​2 mm ​× ​0.5 mm) contains 100 bismuth telluride-based crystals of thermoelectric material (0.17 mm ​× ​0.17 mm ​× ​0.4 mm). This micromodule is installed between two thin Al_2_O_3_-based ceramic plates (diameter 3 mm, thickness 0.1 mm) and carefully sealed from the sides. The developed HF sensor is 3 mm in diameter and 0.7 mm in thickness (contact surface area of about 7.1 mm^2^).

The thermoelectric HF sensor, upon contact with biological tissue, generates a voltage that is accurately measured by the digital microcontroller and converted into a physical quantity expressed in units of HF density (mW/cm^2^). The measurement range of the HF density of the developed device is 0.01 mW/cm^2^ to 50 mW/cm^2^.

**Fig. 1:**
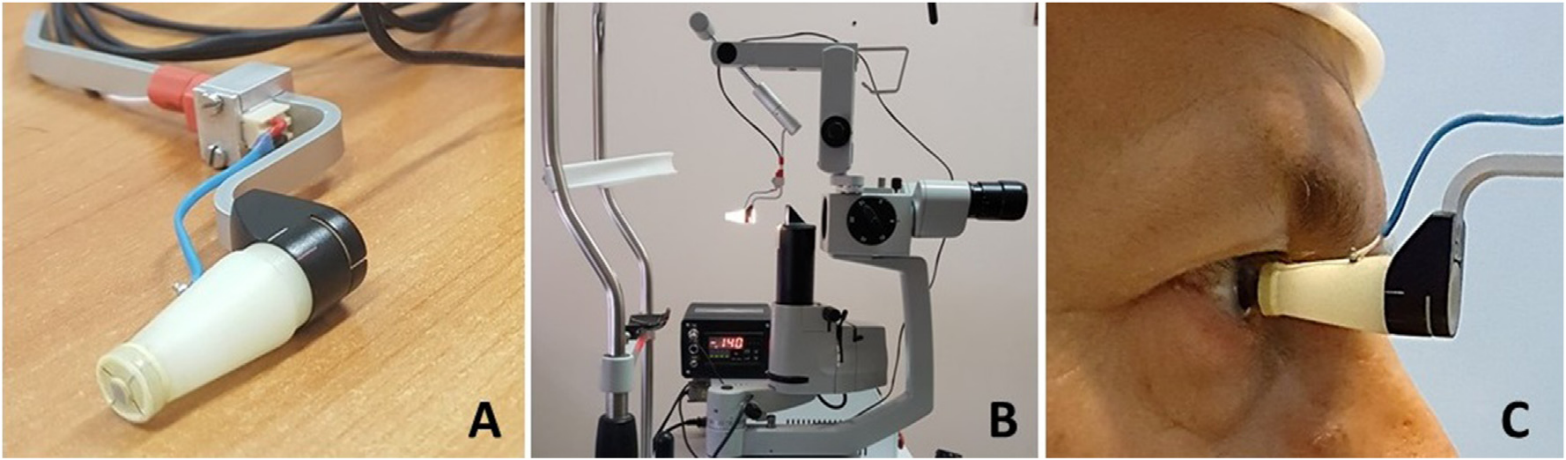
The device for direct measuring of the ocular surface temperature and the HF density. The HF sensor is attached to a specially-made contact prism (A). The prism-sensor unit is attached to the Goldman tonometer's conventional prism mount. The device is attached to the slit lamp (B); The contact surface of the thermoelectric HF sensor is in contact with the human cornea during measurements (C).

The HF sensor is connected to a specially-made contact prism, that can be attached to the standard prism holder of the Goldmann tonometer. The prism-sensor unit is easily attached to a slit lamp. The sample of the device is shown in Fig. 1.

**Fig. 2:**
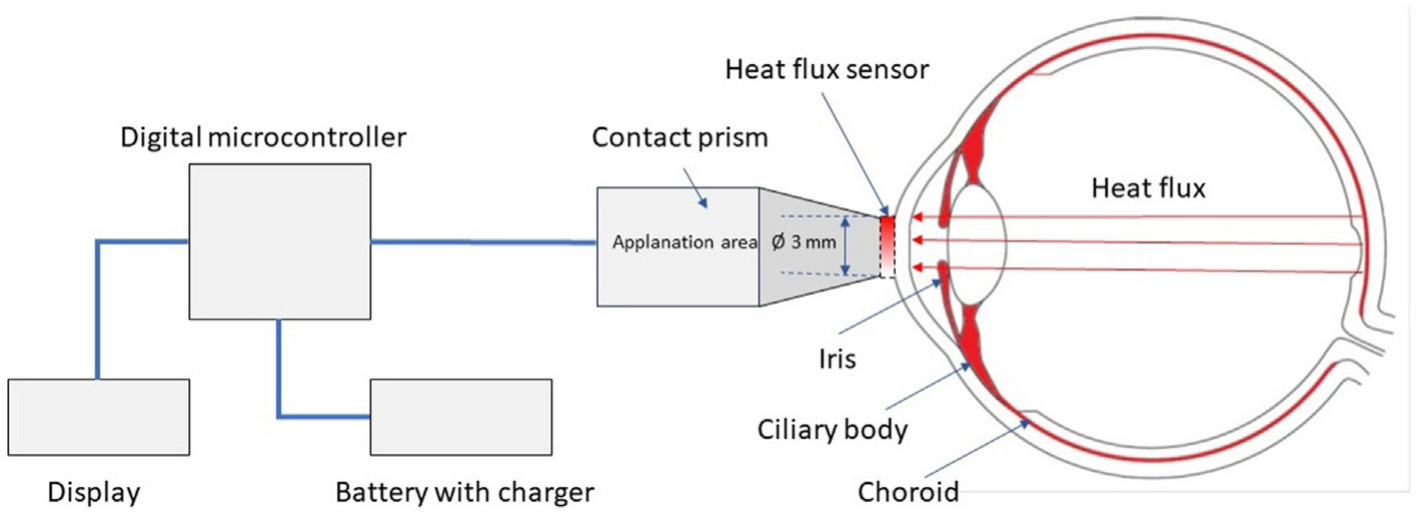
Schematic representation of the device for measurement of the HF density from the ocular surface.

The thermoelectric HF sensor is centred in the contact prism and contacts the eye surface during the examination (in our study, with the central surface of the cornea). A schematic representation of the location of the prism-sensor unit concerning the ocular structures during examination is shown in Fig. 2.

The HF sensor's outer surface is designed to be non-traumatic for the ocular structures. In addition, the prism-sensor unit can be easily disinfected after each examination.

The electronic recording unit includes additional temperature measurement channels: 1) for accurate measurement of the temperature of biological tissues with a thermocouple transducer; 2) for measuring the ambient temperature. The resolution of the temperature measurement of the developed device is 0.01°С. The device is battery-powered.

### 1.3 Measurement technique

A bilateral ophthalmic examination of the ocular surface temperature and HF density were included at baseline. Thermal measurements were carried out during the specified period of the day (from 15:00 to 16:00). The ambient temperature and air humidity were monitored. The room's air velocity was kept at a minimum. The temperature and HF measurements were preceded by the adaptation of the patients to the environmental conditions in the room for 30 min. All DR patients and healthy persons were subjected to epibulbar anaesthesia with a single instillation of an ophthalmic 0.5% solution of proparacaine hydrochloride in both eyes 15 min before the measurements.

The patients were seated in front of a slit lamp so that the eye was located opposite the contact prism with the thermoelectric HF sensor of the device. In all cases, the thermoelectric HF sensor's outer surface was in contact with the surface of the central area of the patient's cornea during measurements.

The ocular surface temperature and HF density were measured again according to the above algorithm 30 min after dilating the patient's pupils with a single instillation of 1% tropicamide solution. In all DR patients, only the pupils of their right eyes were dilated. The left eye was used as the control. Before taking the measurements, all DR patients received a single instillation of a 0.5% solution of proparacaine hydrochloride in both eyes to induce epibulbar anaesthesia. The instillation was administered 15 min before the measurements were taken.

The cornea was stained with fluorescein after the examination. The patient's body temperature and indoor temperature were recorded in all cases.

### 1.4 Statistical analysis

The measurement results were expressed as means (M) ​± ​standard deviations (SD). Categorical data were shown as percentages and frequencies, %(n). Categorical data were analyzed using a chi-square test. To compare HF density (temperature) before and after dilatation, a paired Student's t-test was used. Pearson correlation was used to evaluate the independent correlation between the ocular surface HF density and the corneal temperature, the patient's age, and the thickness of the choroid. The receiver operating characteristic (ROC) curve was plotted and sensitivity and specificity were calculated for the assessment of diagnostic accuracy of the ocular surface HF density test in predicting the presence of proliferative DR. The threshold for statistical significance was established as *P* ​< ​0.05. Statistical analysis was performed using Statistica 10.0 (StatSoft, Tulsa, OK, USA) software.

## 2 Results

The mean room temperature during examination sessions was 22.3 ​± ​0.7°С. The mean temperature of the ocular surface and mean HF density in DR patients were 34.7 ​± ​0.7°С (from 33.5°С to 35.5°С) and 7.2 ​± ​1.3 mW/cm^2^ (from 4.7 mW/cm^2^ to 9.0 mW/cm^2^), respectively. The body temperature of DR patients was recorded at an average of 36.3 ​± ​0.7°C. The OST was positively correlated with the HF density of the eye (r ​= ​0.36; *P* ​= ​0.005) of DR patients. The OST of DR patients (34.7 ​± ​0.7°С) did not differ significantly from the OST of healthy individuals (34.9 ​± ​0.7°C; *P* ​= ​0.7). The HF density of the eyes of DR patients (7.2 ​± ​1.3 mW/cm^2^) was significantly lower compared to healthy individuals (7.8 ​± ​1.2 mW/cm^2^; *P* ​= ​0.002).

The temperature of the ocular surface did not differ significantly in the right and left eyes, as well as the HF density in paired eyes in DR patients and healthy individuals. The ocular surface temperature and the HF density of the right and left eyes of DR patients are presented in Table 1.

**Table 1:** The ocular surface temperature and the HF density of the right and left eyes of DR patients.

	Right eyes, n ​= ​84	Left eyes, n ​= ​84	Significance level
The temperature of the ocular surface, °С	34.6 ​± ​0.7	34.7 ​± ​0.9	*P* ​= ​0.4
The heat flux density of the eye, mW/cm^2^	7.0 ​± ​1.5	7.2 ​± ​1.1	*P* ​= ​0.3

Note: the table shows average values and their standard deviations (M ​± ​SD).

In the following stage, we examined how the density of HF on the ocular surface varies with the age of patients with DR. We found that the ocular HF density was negatively correlated with age in DR patients (r ​= ​- 0.5; *P* ​= ​0.001). All DR patients were divided into three age groups: group 1 (30 participants) aged 18–30 years; group 2 (27 participants) aged 31–60 years; group 3 (27 participants) aged 61–88 years. The ocular surface temperature and the HF density in three age groups of DR patients are presented in Table 2.

**Table 2:** The ocular surface temperature and the HF density in patients with DR in three age groups.

	group 1 aged 18–30 years, n ​= ​30	group 2 aged 31–60 years, n ​= ​27	group 3 aged 61–88 years, n ​= ​27
The temperature of the ocular surface, °С	34.7 ​± ​0.8	34.4 ​± ​0.7	34.2 ​± ​0.7∗
The heat flux density of the eye, mW/cm^2^	8.1 ​± ​1.5	7.2 ​± ​1.7∗	6.2 ​± ​1.8∗^+^

Note: the table shows average values and their standard deviations (M ​± ​SD). ∗, significant difference (*P* ​< ​0.05) to group 1; ^+^, significant difference (*P* ​< ​0.05) compared to group 2.

The mean age of patients with DR (47 ​± ​7 years) and healthy individuals (45 ​± ​8 years) in our study did not differ significantly (*P* ​= ​0.1). Comparison of ocular HF density values in three age groups (group 1, 18–30 years; group 2, 31–60 years; group 3, over 61 years old) in patients with DR and healthy individuals showed: HF density from DR eyes (8.1 ​± ​1.5 mW/cm^2^) did not differ significantly from the ocular HF density of healthy individuals (8.6 ​± ​1.4 mW/cm^2^; *P* ​= ​0.2) in the first age group; HF density of DR eyes of the second (7.2 ​± ​1.7 mW/cm^2^) and third (6.2 ​± ​1.8 mW/cm^2^) age groups were significantly lower compared to healthy individuals of the same age (7.9 ​± ​1.1 mW/cm^2^; *P* ​= ​0.03) and (7.3 ​± ​1.0 mW/cm^2^; *P* ​= ​0.01) respectively.

The duration of diabetes in age group 1 of DR patients was 12 ​± ​7 years, 13 ​± ​9 years (compared to group 1; *P* ​= ​0.6) in group 2, 16 ​± ​7 years (compared to groups 1 and 2; *P* ​= ​0.2 and 0.04 respectively) in group 3.

The thickness of the choroid in DR patients averaged 285 ​± ​54 μm. There was a positive correlation between the thickness of the choroid and the ocular surface HF density (r ​= ​0.6; *P* ​= ​0.001) in contrast to the corneal temperature (r ​= ​0.3; *P* ​= ​0.1). The thickness of the choroid in age group 1 of patients with DR was 298 ​± ​45 μm, in group 2–281 ​± ​55 μm (compared to group 1; *P* ​= ​0.2), and in group 3–273 ​± ​38 μm (compared to groups 1 and 2; *P* ​= ​0.03 and 0.5 respectively).

The values of OST and the HF density in DR patients did not differ significantly depending on gender (*P* ​= ​0.6), type of diabetes (*P* ​= ​0,3), history of COVID-19 (*P* ​= ​0.3), pseudophakia (*P* ​= ​0.2), the presence of macular oedema (*P* ​= ​0.3), and previous intravitreal antiangiogenic therapy (*P* ​= ​0.6).

Forty patients (80 eyes) had bilateral non-proliferative DR, while 44 patients (88 eyes) had bilateral proliferative DR. The baseline OST, HF density, and other demographic and clinical data for patients with non-proliferative and proliferative DR are presented in Table 3.

**Table 3:** Baseline demographic and clinical parameters of patients under study in the non-proliferative DR group and in the proliferative DR group.

Parameters	Non-proliferative DR group, n ​= ​80	Proliferative DR group, n ​= ​88	Significance level, *P*
Age, years	46 ​± ​7	48 ​± ​9	*P* ​= ​0.1
Sex (females), n (%)	48 (60%)	56 (64%)	*P* ​= ​0.6
Type 1 diabetes, n (%)	6 (7.5%)	8 (9%)	*P* ​= ​0.8
Duration of diabetes, years	12 ​± ​8	14 ​± ​7	*P* ​= ​0.1
History of COVID-19, n (%)	32 (40%)	42 (48%)	*P* ​= ​0.4
Number of phacik eyes, n (%)	54 (68%)	61 (69%)	*P* ​= ​0.9
Number of eyes with rubeosis, n (%)	–	21 (24%)	
Number of eyes with macular oedema, n (%)	44 (55%)	53 (60%)	*P* ​= ​0.5
Number of eyes with intravitreal antiangiogenic therapy, n (%)	30 (38%)	39 (44%)	*P* ​= ​0.4
Number of eyes with PRP, n (%)	–	45 (51%)	
Mean IOP, mmHg.	16.4 ​± ​2.1	16.7 ​± ​3.2	*P* ​= ​0.5
Mean retinal thickness, μm.	388 ​± ​121	354 ​± ​140	*P* ​= ​0.1
Mean choroidal thickness, μm.	305 ​± ​75	264 ​± ​96	*P* ​= ​0.003
Mean temperature of the ocular surface, °С	34.8 ​± ​0.8	34.5 ​± ​1.1	*P* ​= ​0.06
Mean ocular HF density, mW/cm^2^	7.3 ​± ​1.2	6.5 ​± ​1.6	*P* ​= ​0.001

Note: the table shows average values and their standard deviations (M ​± ​SD). IOP intraocular pressure, PRP pan-retinal photocoagulation.

The OST of patients with non-proliferative (34.8 ​± ​0.8°C) and proliferative DR (34.5 ​± ​1.1°C) did not differ significantly from the OST of healthy individuals (*P* ​= ​0.8 and *P* ​= ​0.4 respectively). The HF density values of eyes with non-proliferative DR (7.3 ​± ​1.2 mW/cm^2^) also did not differ significantly from the HF density values of the eyes of healthy individuals (7.8 ​± ​1.2 mW/cm^2^; *P* ​= ​0.2). At the same time, the HF density of eyes with proliferative DR (6.5 ​± ​1.6 mW/cm^2^) was significantly lower compared to healthy individuals (*P* ​= ​0.001).

**Fig. 3:**
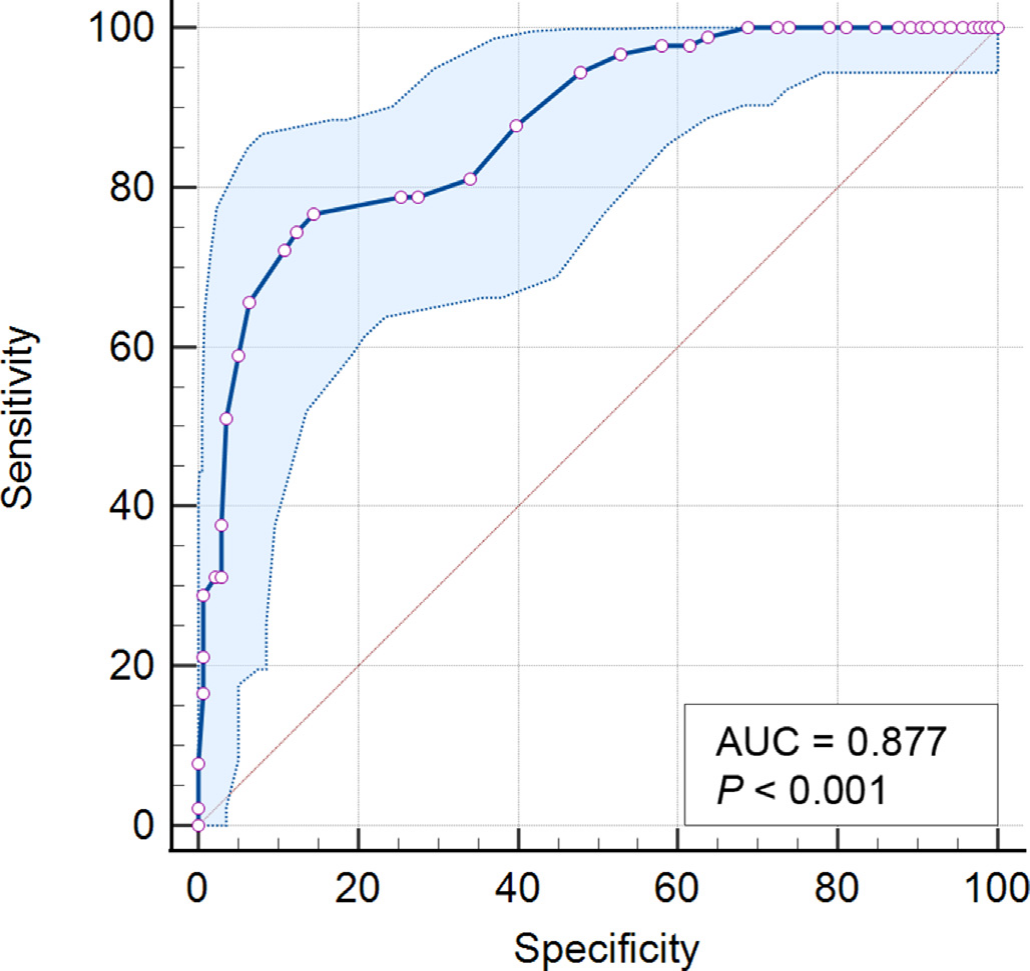
The ROC curve for proliferative DR determining with the ocular surface HF density test (76.67% sensitivity and 85.51% specificity, with an area under curve of 0.877).

The ROC curve for proliferative DR determining with the ocular surface HF density test is shown in Fig. 3. The ROC area under the curve (AUC) for the ocular surface HF density was 0.877 (95% CI, 0.827–0.916). A cut-off value of 6.8 mW/cm^2^ for the ocular surface HF density showed a sensitivity of 76.67% and specificity of 85.51% for detecting proliferative DR.

The ocular surface HF density in eyes with proliferative DR did not differ significantly depending on previous pan-retinal photocoagulation (*P* ​= ​0.6).

Mean values of HF density on the ocular surface in proliferative DR with rubeosis (5.9 ​± ​1.6 mW/cm^2^) were significantly lower than those without rubeosis (6.8 ​± ​1.2 mW/cm^2^; *P* ​= ​0.007).

After the dilation, the surface HF density of the right eyes of DR patients increased significantly compared to the baseline, in contrast to the OST, as presented in Table 4.

**Table 4:** The ocular surface temperature and the HF density of the right eyes of DR patients before and after dilation.

	Right eyes before dilation	Right eyes after dilation	Significance level
The temperature of the ocular surface, °С	34.6 ​± ​0.7	34.9 ​± ​0.8	*P* ​= ​0.08
The heat flux density of the eye, mW/cm^2^	7.0 ​± ​1.5	8.8 ​± ​1.7	*P* ​= ​0.0001

Note: the table shows average values and their standard deviations (M ​± ​SD).

After the dilatation of the right eyes of DR patients, an asymmetry in the HF density of the paired eyes appeared, as presented in Table 5.

**Table 5:** The ocular surface temperature and the HF density of the right eyes after dilation and control left eyes of DR patients.

	Right eyes (after dilation)	Left eyes (control)	Significance level
The temperature of the ocular surface, °С	34.9 ​± ​0.8	34.8 ​± ​0.9	*P* ​= ​0.4
The heat flux density of the eye, mW/cm^2^	8.8 ​± ​1.7	7.6 ​± ​1.4	*P* ​= ​0.0001

Note: the table shows average values and their standard deviations (M ​± ​SD).

Direct real-time measurement of the ocular surface НF density required 40–60 seconds to examine one eye. In all cases, the fluorescein dye test was negative after the measurements, and no changes in the cornea were detected immediately after the examination.

## 3 Discussion

In our study, in DR patients, a tendency to decrease the values of OST and HF density with age was found, which is consistent with the data obtained in healthy individuals.^13^^,^^15^ Subfoveal choroidal thickness was lower in DR patients over 61 years compared with younger patients. Presumably, this may be due to age-related atrophic changes in the choroid.^16^ In addition, we found that patients in the older age group had a longer duration of diabetes than younger patients, which may also influence the choroidal thickness. Ambiya and colleagues^17^ reported that subfoveal choroidal thickness decreases with the increasing duration of diabetes. It is known that the choroid is considered the main source of heat in the human eye and plays an important role in the appearance of the HF of the eye.^6^^,^^12^^,^^18^ Consequently, in patients with DR, a decrease in the thickness of the choroid (due to its age-related and diabetic changes) likely reduces the intensity of ocular heat transfer and leads to a decrease in the HF density on the ocular surface. The detected significant differences in HF density compared to controls in the second and third age groups of DR patients, in contrast to the first group, are presumably also associated with a possible decrease in choroidal heat transfer not only due to the increasing age of patients but also the increasing duration of diabetes.

The assumption about the key role of the choroid in the ocular heat transfer is confirmed by the positive correlation between the thickness of the choroid and the density of HF on the surface of the eye in DR patients, in contrast to corneal temperature. In healthy individuals, the ocular surface HF density also correlates with choroidal blood flow and thickness.^14^

The values of ocular surface HF density in proliferative DR were significantly lower compared to non-proliferative DR. This may also be associated with disorders of choroidal hemodynamics in patients with proliferative DR. The results obtained are consistent with the data we found and the data of other authors that the choroid of the eyes with proliferative DR is much thinner compared to nonproliferative DR.^19^ At the same time, we did not observe that OST differs significantly in patients with proliferative and nonproliferative DR. Obviously, for a comprehensive assessment of the ocular heat transfer, it is advisable to measure both the HF density and OST. The detected ROC AUC values indicate that ocular surface HF density has a high performance in predicting the presence of proliferative DR.

In addition, in patients with proliferative DR, we found lower values of HF density on the surface of eyes with rubeosis compared to eyes without rubeosis. This is likely caused by a deeper disturbance in blood flow and severe tissue ischemia in patients with proliferative DR and rubeosis. These results may be useful in identifying patients with proliferative DR and severe intraocular blood flow insufficiency associated with a high risk of developing neovascular glaucoma.

Previously, no significant interocular differences in OST have been reported in humans.^20^ Morgan and colleagues^21^ reported that in 95 % of the normal population, the difference in interocular OST is no more than 0.6°C. In our study, the temperature and HF of the ocular surface in paired eyes of patients with DR did not differ significantly. This is probably due to the presence of the same stage of DR in both eyes in patients. However, after unilateral pupil dilation in patients with DR, there was no relative equality of the HF density on the surface of the paired eyes.

Chandrasekar and colleagues have shown that pupil dilation increases OST in both healthy individuals and DR patients.^11^ In our study, after pupil dilation, a significant increase in ocular surface HF density was found in DR patients compared to baseline, in contrast to corneal temperature, which is in line with our previous studies in rabbits.^12^ This further confirms that choroidal blood flow is a major contributor to heat transfer in the eye compared to iris and ciliary body circulation. Probably, the iris partially blocks heat transfer from the posterior pole due to less intense blood flow (compared to the choroid) and the relatively low temperature of the aqueous humour.^22^

The limitation of this study is the inclusion of both naive DR patients and patients previously treated with intravitreal antiangiogenic therapy and pan-retinal photocoagulation. At the same time, there were no significant differences in the ocular surface temperature and the HF density in DR patients with a history of pan-retinal photocoagulation or intravitreal antiangiogenic therapy.

The device we used to measure ocular surface HF density requires contact with the cornea. The technique for measuring HF density of the ocular surface is similar to Goldmann applanation tonometry, which is known as the gold standard for measuring intraocular pressure.^23^ At the same time, contact ocular examination may be accompanied by a risk of corneal damage in some cases.^24^ In our study, no corneal damage was found after measurements and was confirmed by a negative test with fluorescein staining. The use of the device has shown that it is safe and allows convenient measurement of HF density on the ocular surface.

## 4 Conclusions

We believe that measurements of the ocular surface HF density in patients with DR can be used as a physiologic biomarker of intraocular blood flow insufficiency. Direct measurement of ocular surface HF density may be useful for the early detection of DR patients with severe ocular tissue ischemia and a high risk of neovascular glaucoma. Additional studies will be required to assess changes in ocular HF values in patients with DR after different types of treatment.

## Study approval

The authors confirm that any aspect of the work covered in this manuscript that involved human patients or animals was conducted with the ethical approval of all relevant bodies and the ​study was performed in accordance with the Declaration of Helsinki.

## Author contributions

The authors confirm their contribution to the paper as follows: Conception and design of the study: LA, NP; Data collection: OZ, RK, TK, IN, AK; Analysis and interpretation of results: OZ, AK; Drafting the manuscript: OZ. All authors reviewed the results and approved the final version of the manuscript.

## Funding

This research did not receive any specific grant from funding agencies in the public, commercial, or not-for-profit sectors.

## Declaration of competing interest

The authors declare that they have no known competing financial interests or personal relationships that could have appeared to influence the work reported in this paper.
